# Identification of Iridoids in Edible Honeysuckle Berries (*Lonicera caerulea* L. var. *kamtschatica* Sevast.) by UPLC-ESI-qTOF-MS/MS

**DOI:** 10.3390/molecules21091157

**Published:** 2016-09-01

**Authors:** Alicja Z. Kucharska, Izabela Fecka

**Affiliations:** 1 Department of Fruit and Vegetables and Cereals Technology, Wrocław University of Environmental and Life Science, Chełmońskiego 37, 51-630 Wrocław, Poland; 2 Department of Pharmacognosy, Wrocław Medical University, Borowska 211A, 50-556 Wrocław, Poland; izabela.fecka@umed.wroc.pl

**Keywords:** iridoids, loganin 7-*O*-pentoside, *epi*-isomers, honeysuckle berries, LC-ESI-qTOF-MS/MS, fragmentation pathway

## Abstract

Iridoid profiles of honeysuckle berry were studied. Compounds were identified by ultra-performance liquid chromatography coupled with electrospray ionization mass spectrometry UPLC-ESI-qTOF-MS/MS in positive and negative ions mode. The MS fragmentation pathways of detected iridoid glycosides were also studied in both modes. In the negative ESI mass spectra, iridoids with a methyl ester or lactone structure have preferentially produced adduct [M + HCOOH − H]^−^ ions. However, protonated ions of molecular fragments, which were released by glycosidic bond cleavage and following fragmentation of aglycone rings, were more usable for iridoid structure analysis. In addition, the neutral losses of H_2_O, CO, CO_2_, CH_3_OH, acetylene, ethenone and cyclopropynone have provided data confirming the presence of functional substituents in the aglycone. Among the 13 iridoids, 11 were identified in honeysuckle berries for the first time: pentosides of loganic acid (two isomers), pentosides of loganin (three isomers), pentosyl sweroside, and additionally 7-*epi*-loganic acid, 7-*epi*-loganin, sweroside, secologanin, and secoxyloganin. The five pentoside derivatives of loganic acid and loganin have not been previously detected in the analyzed species. Honeysuckle berries are a source of iridoids with different structures, compounds that are rarely present in fruits.

## 1. Introduction

*Lonicera caerulea* L. var. *kamtschatica* Sevast. (Caprifoliaceae) is a fruit bush originating from the Far East of Russia (Kamchatka Peninsula). Edible honeysuckle berries are gaining popularity in many European countries, Japan, China, Canada and others. These berries are good fresh fruit for consumption and good raw material for the production of juices, snacks, dry fruits, and jams [1,2,3]. Anthocyanins, flavan-3-ols, flavonols, phenolic acids, and vitamin C are the main compositional groups in honeysuckle berries that exhibit antioxidant, antibacterial, antiviral and anti-inflammatory effects [1,3,4,5,6,7,8]. Honeysuckle berries’ health benefits have been known and used for a long time in traditional medicine in Russia and China.

Iridoids are a large group of secondary metabolites found both in a variety of plant and selected animal species. They belong to the monoterpenes with a cyclopentanopyran skeleton and occur in plant materials naturally as glucoside forms. Iridoids, depending on the chemical structure, exhibit different pharmacological properties, such as antibiotic, anti-inflammatory or hypertensive activities [9]. They have varying degrees of bitterness. Especially known in this respect are secoiridoids, which also exhibit the feature of deterrence of herbivores. The secoiridoids are a subclass of iridoids with an opening of the cyclopentane ring between the carbon atoms C-7 and C-8. A large variety of iridoid structures exists. To identify them, particularly in complex plant extracts, the LC-MS method is the most useful, especially when coupled to a soft ionization source, e.g., electrospray ionization (ESI). Quadrupole time-of-flight (QTOF) MS/MS allows one to measure mass with high accuracy and to track fragmentation of ions, which enables good interpretation of results, contributing to determination of the empirical chemical formulas. Many authors successfully identify iridoids in raw plants by this method. Some authors have analyzed iridoids in negative mode [10,11,12], others in positive mode [13], the mode used depending on the structure of chemical compounds. 

Iridoids, in contrast to polyphenols, are rarely found in fruits, but they are found in green parts of plants. Exceptions are, e.g., cornelian cherry fruits [14,15]. Among the species of the genus *Lonicera*, iridoids have been identified mainly in leaves of *L. caerulea* [16,17] and in flowers, buds, stem, leaves, and caulis of *L. japonica* [12,13,18,19]. There are few reports on iridoid contents in fruits of *Lonicera*. Whitehead and Bowers [20] determined six compounds from this group in non-edible fruits from the species *Lonicera morrowii* A. Gray, *Lonicera tatarica* L., and their hybrid *Lonicera* × *bella* Zabel. In the edible fruits from *L. caerulea*, only one bitter iridoid was identified—7-oxologanin [21]. To our knowledge, there are no studies available on iridoids in berries from the species *L. caerulea* var. *kamtschatica*, except our own previous conference reports [14,22]. Therefore, the aim of this study was to present a complete qualitative determination of iridoids in berries of blue honeysuckle varieties (*L*. *caerulea* var. *kamtschatica*). This is the first detailed paper about iridoid composition of blue honeysuckle berries.

## 2. Results and Discussion

**Figure 1:**
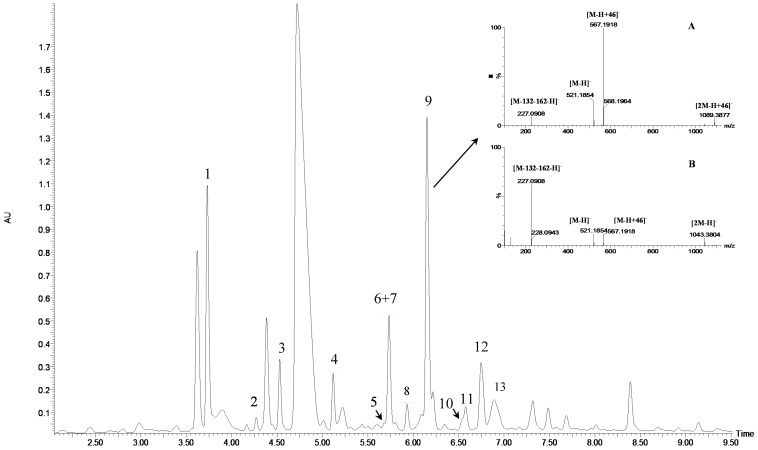
UPLC-DAD chromatogram (254 nm) of compounds of methanolic extract from honeysuckle berries (*Lonicera caerulea* L.) and mass spectra of loganin 7-*O*-pentoside (peak 9) before (**A**) and after (**B**) fragmentations in negative mode. The peak number corresponds to the number in Table 1.

The acidified methanolic extract of honeysuckle berries was analyzed by the UPLC-qTOF-MS/MS method. The compounds were identified by their UPLC retention times, elution order, UV-Vis (200–600 nm) and MS spectra, and by comparison with available standards and literature data. The UPLC chromatogram of honeysuckle berry extract, obtained in the UV spectral region (254 nm), is shown in Figure 1. UV-Vis spectra of 13 compounds indicate that they are iridoids. In our previous initial studies, we determined five iridoids (loganic acid, loganin and their three derivatives) from honeysuckle berries [14,22], but we could not completely prove their structures. In this study, 13 iridoids were exactly identified by LC-MS/MS; among these, eight compounds were discovered for the first time.

**Figure 2:**
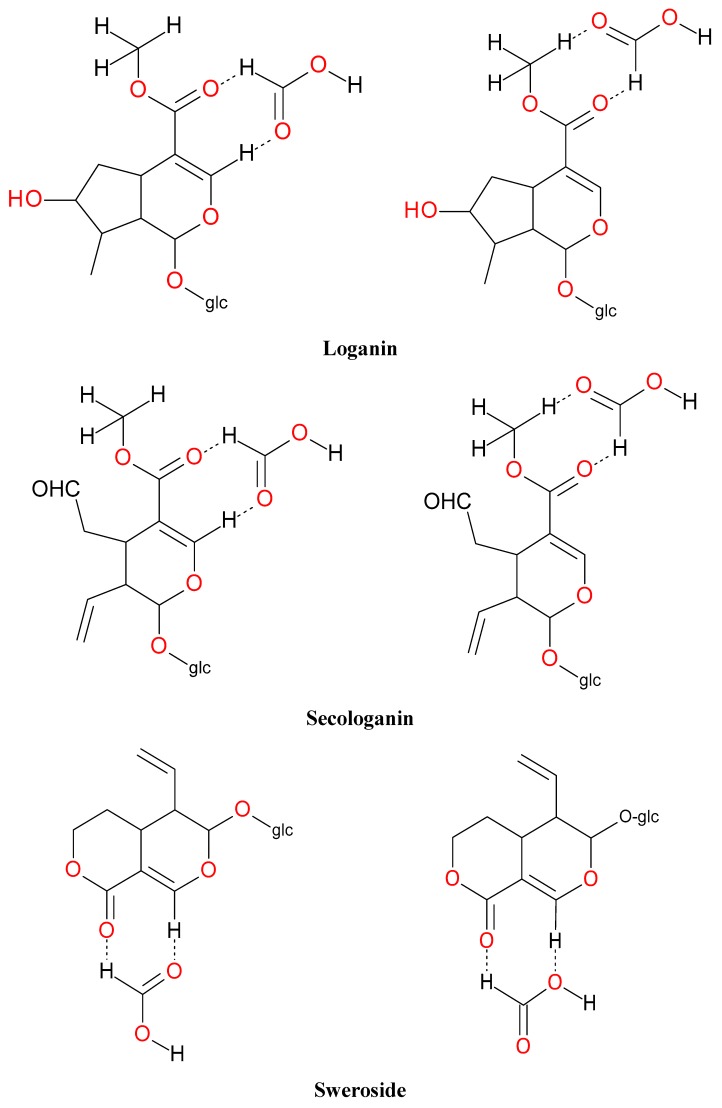
Loganin, secologanin, and sweroside with formic acid possible adducts.

Iridoids with methyl ester or lactone structure showed a strong tendency for associated product ion formation in negative ionizations; therefore MS data of ions of these and other compounds, in both negative and positive ionization modes, were investigated (Table 1). In the literature, those associated product ions are commonly called adducts [10,11,19]. Depending on the acid applied in the mobile phase, formic acid [11] or acetic acid [19] adducts may appear. Under our analysis conditions, possible associations of loganin, secologanin and sweroside with formic acid connected by hydrogen bonds are shown in Figure 2. However, in the case of secoxyloganin, which possesses a methyl ester and additionally a free carboxyl group, we did not observe a similar phenomenon. The reason for this difference may be mutual influence of the discussed groups resulting in internal weak hydrogen bonds, preventing formation of an association with formic acid.

The results of LC-MS/MS analysis of four compounds (peaks 1, 2, 7, and 10) were compared to the results of the available iridoid standards. The compounds **1** and **2** (*t*_R_ 3.73 min, and *t*_R_ 4.27 min), and the loganic acid standard have a molecular ion at *m*/*z* 375.1276 [M − H]^−^ and a fragment ion at *m*/*z* 213.0769 [M − 162 − H]^−^ in negative ESI mode, and a pseudomolecular ion at *m*/*z* 377.1440 [M + H]^+^ and a fragment ion at *m*/*z* 215.0913 [M − 162 + H]^+^ in positive ESI mode (Table 1). Compounds **7** and **10** (*t*_R_ 5.73 min, *t*_R_ 6.54 min) and the loganin standard displayed a formic acid associated ion at *m*/*z* 435.1502 [M + 46 − H]^−^ and a fragment ion at *m*/*z* 227.0939 [M − 162 − H]^−^ in negative ESI mode, and a pseudomolecular ion at *m*/*z* 391.1554 [M + H]^+^ and a fragment ion at *m*/*z* 229.1062 [M − 162 + H]^+^ in positive ESI mode.

**Figure 3:**
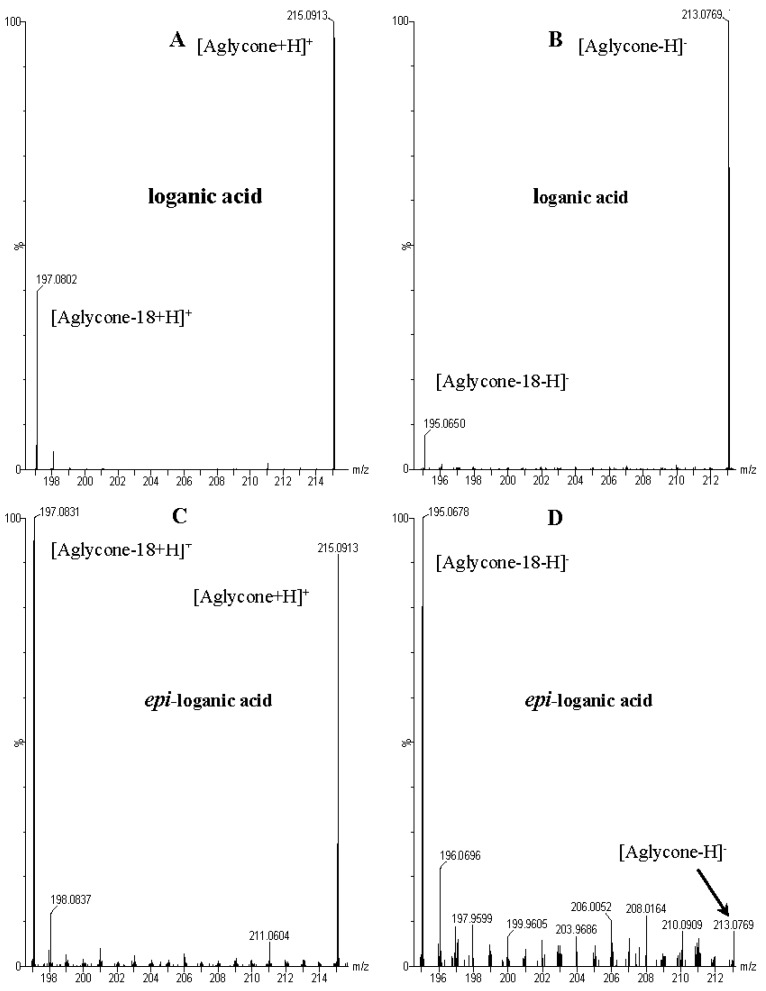
Mass spectra of the aglycone ions of loganic acid (1) and *epi*-loganic acid (2) after fragmentation in positive (**A**,**C**) and negative (**B**,**D**) mode.

**Figure 4:**
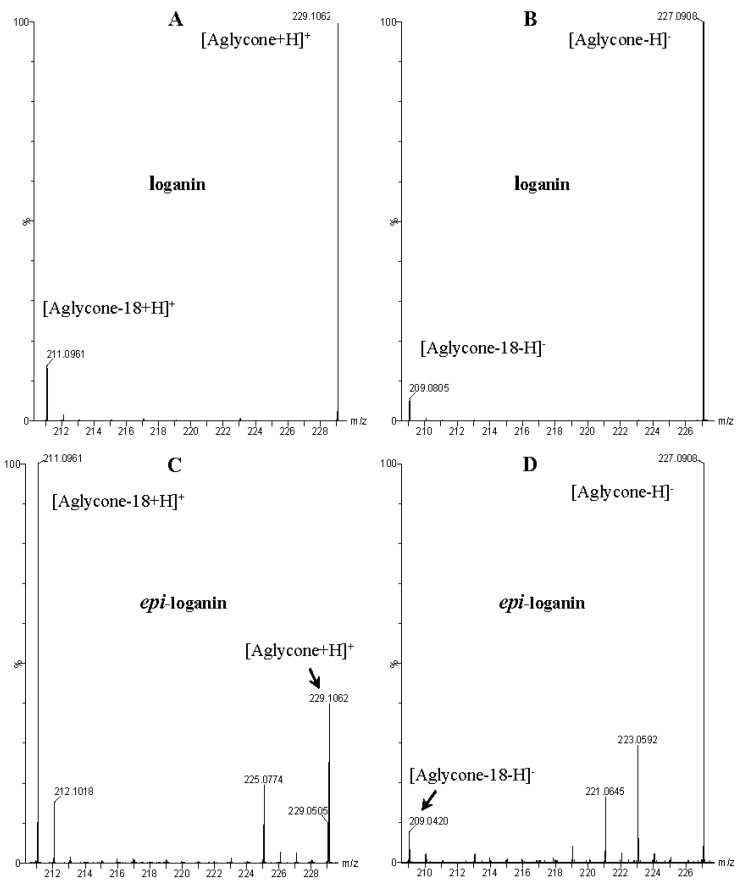
Mass spectra of the aglycone ions of loganin (**7**) and *epi*-loganin (**10**) after fragmentation in positive (**A**,**C**) and negative (**B**,**D**) mode.

These data, UV-Vis absorption spectra and retention times show that compounds **1** and **7** are loganic acid and loganin, respectively. Identification of these compounds was consistent with our previous studies [14,22]. Compounds **2** and **10** displayed the same pseudomolecular and fragment ions in negative and positive mode as compounds **1** and **7**, respectively, but they differed in abundance of the major fragment ions [M − 162 + H]^+^ and [M − 162 − 18 + H]^+^. In compound **1** and loganic acid standard, higher abundance was observed for ions at *m*/*z* 213.0769 [M − 162 − H]^−^ and 215.0913 [M − 162 + H]^+^, while in compound **2** higher abundance was observed for ions at *m*/*z* 195.0678 [M − 162 − 18 − H]^−^ and 197.0831 [M − 162 − 18 + H]^+^ (Figure 3). This indicates that compound **2** is 7-*epi*-loganic acid. Similar differences were observed in the case of compounds **10** and **7**, but only in positive mode. In compound **7** and in loganin standard, higher abundance was detected for the ion at *m*/*z* 229.1062 [M − 162 + H]^+^ and in compound **10** for the ion at *m*/*z* 211.0961 [M − 162 − 18 + H]^+^ (Figure 4). This indicates that compound **10** is 7-*epi*-loganin. Ion [M − 162 + H]^+^ is more stable when the –OH group in C-7 is above the molecule plane. Conversely, when the –OH group is below the plane, this ion is less stable, or its stabilization is not possible; therefore the ion appears after loss of water (18 Da). Similar observations were made by Madhusudanan et al. [23] for the epimers of loganin after fragmentation in positive mode.

**Figure 5:**
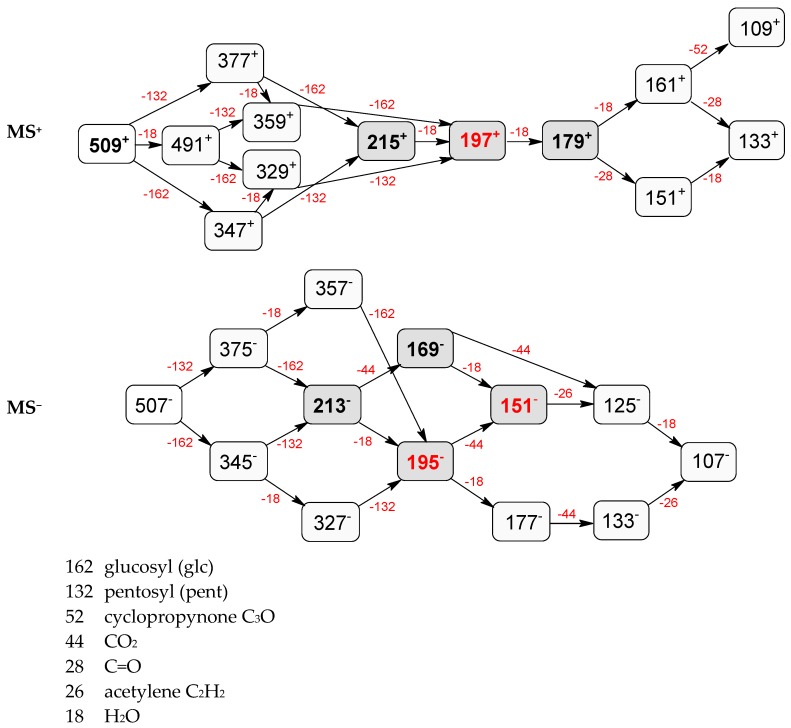
Fragmentation pathway of loganic acid 7-*O*-pentoside (509^+^/507**^−^**), *epi*-loganic 7-*O*-pentoside acid (509^+^/507**^−^**), loganic acid (377^+^/375**^−^**), and *epi*-loganic acid (377^+^/375**^−^**) in the positive (MS^+^) and negative (MS^−^) mode with the dominant ions in form *epi* (**red ions**).

Compounds **3** (*t*_R_ 4.53 min) and **4** (*t*_R_ 5.2 min) also display the same pseudomolecular ions [M − H]^−^ at *m*/*z* 507.1746 and [M + H]^+^ at *m*/*z* 509.1876, and the same fragment ion [M − 132 + H]^+^ at *m*/*z* 377.1493 (Table 1). These ions corresponded to the molecular ion of the loganic acid after loss of 132 Da. The difference between these compounds, in addition to the retention times, is in MS2. Compound **3**, after fragmentation, gave a stable fragment ion [M − 132 − 162 − H]^−^ at *m*/*z* 213.0769 in negative mode, and [M − 132 − 162 + H]^+^ at *m*/*z* 215.0913 in positive mode, whereas compound **4** gave a stable fragment ion, after loss of water − 18 Da, [M − 132 − 162 − 18 − H]^−^ at *m*/*z* 195.0650 in negative mode and [M − 132 − 162 − 18 + H]^+^ at *m*/*z* 197.0802 in positive mode, which is comparable to compounds **1** and **2** (Table 1; Figure 3 and Figure 5). These results suggest that compounds **3** and **4** are pentose derivatives of loganic acid and *epi*-loganic acid, respectively. Fragment ions at *m*/*z* 345.0806 [M − 162 − H]^−^ and 347.1344 [M − 162 + H]^+^ (compound **3**) and 327.1092 [M − 162 − 18 − H]^−^ and 329.1228 [M − 162 − 18 + H]^+^ (compound **4**) show that the pentose is not attached to glucose, and does not form with it a disaccharide. Loss of glucose, while preserving the glycosidic bond of the pentose, indicates that the only possible position to attach pentose to aglycone is at the –OH group in C-7 (Table 2 and Table 3, Figure 5). This proves that compounds **3** and **4** are loganic acid 7-*O*-pentoside and 7-*epi*-loganic acid 7-*O*-pentoside, respectively. The fragmentation pathways of loganic acid and its derivatives in positive and negative mode are shown in Figure 5. The molecular ion at *m*/*z* 509.1876 had two fragmentation pathways: one through preceding loss of water followed by that of the sugars, and conversely the second primarily through loss of sugars followed by that of water. In addition, the neutral losses of CO, CO_2_, acetylene and cyclopropynone have provided data for confirming the presence of functional substituents in the aglycone. All possible secondary structures of monoisotopic ions after negative and positive fragmentations of 7-*O*-pentoside loganic acid, 7-*O*-pentoside *epi*-loganic acid, loganic acid, and *epi*-loganic acid are shown in Table 2 and Table 3.

**Figure 6:**
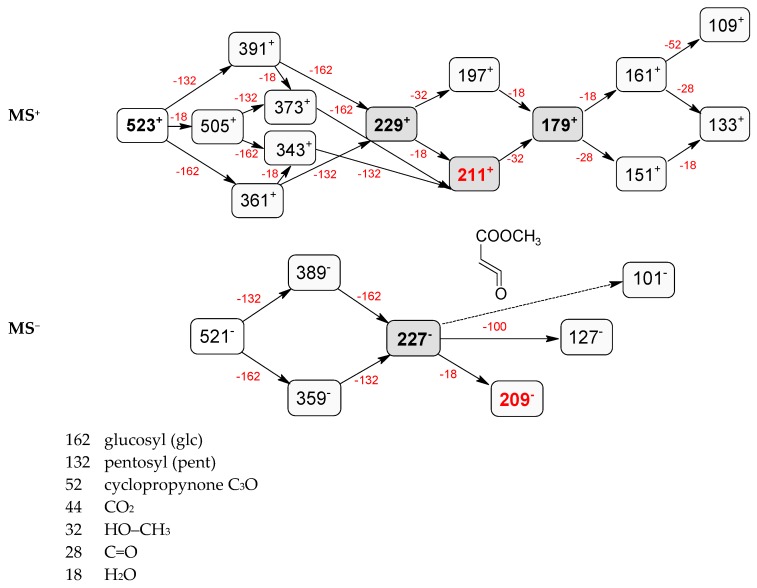
Fragmentation pathway of pentosyl loganin (523^+^/521^−^), loganin 7-*O*-pentoside (523^+^/521^−^), *epi*-loganin 7-*O*-pentoside (523^+^/521^−^), loganin (391^+^/389^−^), and 7-*epi*-loganin (391^+^/389^−^) in the positive (MS^+^) and negative (MS^−^) mode.

Compounds **5**, **6**, **8**, **9**, **12**, and **13**, like loganin (**7**) and *epi*-loganin (**10**), all displayed the formic acid adduct [M + 46 − H]^−^ in negative mode, suggesting that their structures may contain for example the methyl ester functional group or lactone [11]. Similarly, a strong tendency for formation of an acetic adduct was observed by Zhang et al. (2015) for iridoids without a carboxyl group at the mobile phase with acetic acid [19]. Compound **5** (*t*_R_ 5.68 min) showed the same formic acid adduct as compounds **9** (*t*_R_ 6.15 min) and **12** (*t*_R_ 6.78 min) at *m*/*z* 567.1918 [M + 46 − H]^−^ and the same fragment ion at *m*/*z* 227.0939 [M − 132 − 162 − H]^−^ (Table 1; Figure 1). In positive mode, these compounds had a pseudomolecular ion at *m*/*z* 523.2039 [M + H]^+^ and a fragment ion at *m*/*z* 229.1062 [M − 132 − 162 + H]^+^. So these were identified tentatively as pentose derivatives of loganin. Compound **9** displayed the same fragmentation ions [M − 162 + H]^+^ and [M − 162 − 18 + H]^+^ as compound **12** at *m*/*z* 361.1494 and 343.1367, respectively. Compound **5** did not show these ions. According to these data, in compounds **9** and **12**, pentose is attached to aglycone at the –OH group in C-7, whereas in compound **5**, pentose is attached to glucose producing disaccharide (Table 4 and Table 5). Compounds **5** and **9** exhibited a stable fragment ion at *m*/*z* 229.1062 [M − 132 − 162 + H]^+^, whereas compound **12** exhibited a stable ion after loss of water at *m*/*z* 211.0961 [M − 132 − 162 − 18 + H]^+^, which is comparable to compounds **7** and **10** (Figure 4). Further characteristic protonated fragment ions, which were produced after neutral loss of methanol (32 Da) at *m*/*z* 197.0802 and 179.0701 were also detected. Thus, they were pentosyl loganin (**5**), loganin 7-*O*-pentoside (**9**), and 7-*epi*-loganin 7-*O*-pentoside (**12**). The pentose derivatives of loganin, as derivatives of loganic acid, had two fragmentation pathways (Figure 6). In negative ion mode, iridoids with the structure of methyl ester showed weaker ionization, and delivered only a few fragmentation ions. In the case of loganin and 7-*epi*-loganin glycosides, relatively high abundance of ions resulted from free aglycone (*m*/*z* 227.0748) and from two fragments produced as a result of opening the pyran ring from their basic skeleton (*m*/*z* 127.0748 and 101.0252, Table 4).

The most possible structures of monoisotopic ions after negative and positive fragmentations of pentosyl-loganin, loganin 7-*O*-pentoside, 7-*epi*-loganin 7-*O*-pentoside, loganin, and 7-*epi*-loganin are shown in Table 4 and Table 5. The pentose derivatives of both loganic acid and loganin epimers were identified in edible honeysuckle berries for the first time. Additionally, to our knowledge, those compounds were not described in raw materials (plants). In the literature, only apiofuranosyl attached to iridoids, such as sweroside and mussaenosidic acid, has been described [24,25]. Complete elucidation of structures of detected iridoid pentosides, especially glycone (sugar) moieties, will be possible after further studies, including separation of individual compounds from plant material and their spectroscopic analysis.

For compound **13** (*t*_R_ 6.89 min), the formic acid adduct [M + 46 − H]^−^ and fragment ion [M − 162 − H]^−^ were at *m*/*z* 433.1331 and 225.0781, respectively. In positive mode this compound gave an [M + H]^+^ ion at *m*/*z* 389.1433 and a fragment ion at *m*/*z* 227.0920 (aglycone). Between protonated fragment ions, there were also detected signals deriving from structures after loss of ethenone (42 Da, CH_2_=C=O), which was specific for the aldehyde group (–CH_2_–CHO) (Table 1).

According to these data and a previous study [19], this compound was tentatively identified as secologanin. This secoiridoid has been identified not only in *L. japonica* [12] but also in Cornus species [26].

Compound **11** (*t*_R_ 6.58 min) exhibited the pseudomolecular ion [M − H]^−^ at *m*/*z* 403.1263 and the fragment ions at *m*/*z* 371.0952, 223.0622, 179.0568, and 121.0288, which corresponded to secoxyloganin. These data are consistent with the literature [18]. Identification of the compound as secoxyloganin is also confirmed by data in positive mode (Table 1) and the literature data [13].

Compounds **6** (t_R_ 5.73 min) and **8** (*t*_R_ 5.93 min) exhibited the formic acid adducts [M + 46 − H]^−^ at *m*/*z* 403.1223 and 535.1502, respectively (Table 1). Their pseudomolecular ions [M − H]^−^ at *m*/*z* 357.1183 (**6**) and 489.1342 (**8**) in negative mode, and ions [M + H]^+^ at *m*/*z* 359.1347 (**6**) and 491.1775 (**8**) in positive mode, suggest that the difference between these compounds is 132 Da. The loss of a glucose unit (162 Da) in compound **6** and pentose and glucose units (132 Da and 162 Da, respectively) in compound **8** generated the aglycone ion at *m*/*z* 195.0650 and 197.0802 in negative and positive mode, respectively. According to these data and the literature [13,18,19], compound **6** was identified as sweroside, and compound **8** as pentosyl sweroside. 6′-*O*-β-apiofuranosyl sweroside was identified in the ethanolic extract of the roots of *L. quinquelocularis* by Kumar et al. [24].

## 3. Materials and Methods 

### 3.1. Chemicals

Acetonitrile for LC-MS was purchased from POCh (Gliwice, Poland). Formic acid was acquired from Sigma-Aldrich (Steinheim, Germany). Loganic acid and loganin were purchased from Extrasynthese (Lyon Nord, France).

### 3.2. Plant Material

Honeysuckle berries (*Lonicera caerulea* L. var. *kamtschatica* Sevast.) of “Atut” cultivars were used for this study. Fruits were collected from the Research Station for Cultivar Testing in Masłowice and hand-harvested at the stage of consumption during the growing season of May 2014. Before analysis, fruits were frozen and stored at −20 °C.

### 3.3. Extraction of Compounds for Qualitative Analysis

Frozen fruits of honeysuckle were homogenized and 5 g of the homogenate was extracted with 50 mL of 80% aqueous methanol (*v*/*v*) acidified with 1% HCl by ultrasonication for 20 min. The extract was centrifuged and diluted (re-distilled water with the ratio 1:1, *v*/*v*). For UPLC-MS analysis the supernatant was filtered through a Hydrophilic PTFE 0.22 μm membrane (Millex Samplicity Filter, Merck, Darmstadt, Germany) and used for analysis.

### 3.4. Identification of Iridoids by UPLC-qTOF-MS/MS

The method was described previously [27]. Identification of compounds was performed on the Acquity ultra-performance liquid chromatography (UPLC) system, coupled with a quadrupole-time of flight (q-TOF) MS instrument (UPLC/Synapt q-TOF MS, Waters Corp., Milford, MA, USA), with an electrospray ionization (ESI) source. Separation was achieved on the Acquity TM BEH C18 column (100 mm × 2.1 mm i.d., 1.7 µm; Waters). The mobile phase was a mixture of 4.5% aq. formic acid *v*/*v* (A) and acetonitrile (B). The gradient program was as follows: initial conditions—1% B in A, 12 min—25% B in A, 12.5 min—100% B, 13.5 min—1% B in A. The flow rate was 0.45 mL/min and the injection volume was 5 µL. The column was operated at 30 °C. UV-vis absorption spectra were recorded on-line during UPLC analysis, and the spectral measurements were made in the wavelength range of 200–600 nm, in steps of 2 nm. The major operating parameters for the q-TOF MS were set as follows: capillary voltage 2.0 kV, cone voltage 40 V, cone gas flow of 11 L/h, collision energy 28–30 eV, source temperature 100 °C, desolvation temperature 250 °C, collision gas, argon; desolvation gas (nitrogen) flow rate, 600 L/h; data acquisition range, *m*/*z* 100–2000 Da; ionization mode, negative and positive. The data were collected with Mass-Lynx V 4.1 software. The runs were monitored at the wavelength of 254 nm.

## 4. Conclusions 

In this study, 13 iridoids in the methanolic extract from honeysuckle berries were characterized using an UPLC-ESI-qTOF-MS/MS method in positive and negative mode, which can be complementary. The study shows that in honeysuckle berries there is a range of different iridoids, which are rarely present in edible fruits. The use of both ionization modes enabled the elucidation of the structure of analyzed iridoid glycosides, especially the position of pentose substitution and the aglycone stereochemistry. The presence of fragment ions deriving from the secondary pentosides, after the cleavage of glucose, confirmed additional glycosylation of aglycone at the C-7 position (at the –OH group). For diagnostic purposes, more useful data were derived from the MS^+^ spectra, which come from the fragment structures formed after the successive loss of monosaccharide residues and neutral molecules as water (18 Da), CO (28 Da), CO_2_ (44 Da), acetylene (26 Da), methanol (32 Da), ethenone (42 Da) and cyclopropynone (52 Da). It was also observed that the iridoids with the methyl ester structure poorly ionized in the negative ion current, which makes the identification of their epimer pairs particularly difficult. Among the 13 iridoids, 11 were identified for the first time. The identified compounds included pentoside derivatives of loganic acid (two isomers), loganin (three isomers), and sweroside (one compound) and additionally epimeric pairs of loganic acid and loganin, sweroside, secologanin and secoxyloganin. The five pentoside derivatives of loganic acid and loganin have not previously been detected in the analyzed species.

## Figures and Tables

**Table 1:** MS data for identification of iridoids of honeysuckle berries (*Lonicera caerulea* L.).

Peak No.	*t*_R_ (min)	Ion	MS^1^ (*m/z*)	MS^2^ (*m/z*)	Identification
1	3.73	−	375.1276 [M − H]^−^ 751.2630 [2M − H]^−^ 1127.4083 [3M − H]^−^	213.0769 [M − 162 − H]^−^, 195.0650 [M − 162 − 18 − H]^−^, 169.0855 [M − 162 − 44 − H]^−^, 151.0771 [M − 162 − 18 − 44 − H]^−^, 125.0608 [M − 162 − 18 − 44 − 26 − H]^−^, 101.0232	loganic acid
		+	377.1440 [M + H]^+^ 753.2834 [2M + H]^+^	215.0913 [M − 162 + H]^+^, 197.0831 [M − 162 − 18 + H]^+^, 179.0701 [M − 162 − 18 − 18 + H]^+^, 151.0394 [M − 162 − 18 − 18 − 28 + H]^+^, 161.0598 [M − 162 − 18 − 18 − 18 + H]^+^, 133.0659 [M − 162 − 18 − 18 − 28 − 18 + H]^+^, 109.0671 [M − 162 − 18 − 18 − 18 − 52 + H]^+^	
2	4.27	−	375.1276 [M − H]^−^ 751.2630 [2M − H]^−^	213.0769 [M − 162 − H]^−^, 195.0650 [M − 162 − 18 − H]^−^, 169.0855 [M − 162 − 44 − H]^−^, 151.0771 [M − 162 − 44 − 18 − H]^−^, 125.0608 [M − 162 − 18 − 44 − 26 − H]^−^, 01.0232	7-*epi*-loganic acid
		+	377.1440 [M + H]^+^ 753.2834 [2M + H]^+^	215.0913 [M − 162 + H]^+^, 197.0831 [M − 162 − 188 + H]^+^, 179.0701 [M − 162 − 18− 18 + H]^+^, 151.0394 [M − 162 − 18 − 18 − 28 + H]^+^, 161.0598 [M − 162 − 18 − 18 − 18 + H]^+^, 133.0659 [M − 162 − 18 − 18 − 28 − 18 + H]^+^, 109.0671 [M − 162 − 18 − 18 − 18 − 52 + H]^+^	
3	4.53	−	507.1746 [M − H]^−^ 1015.3484 [2M − H]^−^	375.1356 [M − 132 − H]^−^, 345.0806 [M − 162 − H]^−^, 213.0769 [M − 132 − 162 − H]^−^, 195.0650 [M − 162 − 18 − H]^−^, 169.0855 [M − 132 − 162 − 44 − H]^−^, 151.0746 [M − 132 − 162 − 44 − 18 − H]^−^, 125.0608 [M − 132 − 162 − 18 − 44 − 26 − H]^−^, 101.0232	loganic acid 7-*O*-pentoside
		+	509.1876 [M + H]^+^ 1017.3707 [2M + H]^+^	491.1775 [M − 18 + H]^+^, 377.1493 [M − 132 + H]^+^, 359.1348 [M − 18 − 132 + H]^+^, 347.1344 [M − 162 + H]^+^, 329.1228 [M − 18 − 162 + H]^+^, 215.0913 [M − 132 − 162 + H]^+^, 197.0802 [M − 132 − 162 − 18 + H]^+^, 179.0701 [M − 132 − 162 − 18 − 18 + H]^+^, 161.0598 [M − 132 − 162 − 18 − 18 − 18 + H]^+^, 151.0771 [M − 132 − 162 − 18 − 18 − 28 + H]^+^, 133.0659 [M − 132 − 162 − 18 − 18 − 28 − 18 + H]^+^, 109.0671 [M − 132 − 162 − 18 − 18 − 18 − 52 + H]^+^	
4	5.12	−	507.1746 [M − H]^−^ 1015.3484 [2M − H]^−^	357.1144 [M − 132 − 18 − H]^−^, 327.1092 [M − 162 − 18 − H]^−^, 195.0650 [M − 132 − 18 − 162 − H]^−^, 177.0549 [M − 132 − 18 − 162 − 18 − H]^−^, 151.0771 [M − 132 − 18 − 162 − 18 − 44 − H]^−^, 133.0654 [M − 132 − 18 − 162 − 18 − 44 − H]^−^, 125.0608 [M − 132 − 18 − 162 − 44 − 26 − H]^−^ 101.0232	7-*epi*-loganic acid 7-*O*-pentoside
		+	509.1876 [M + H]^+^ 1017.3707 [2M + H]^+^	491.1775 [M − 18 + H]^+^, 377.1493 [M − 132 + H]^+^, 359.1348 [M − 18 − 132 + H]^+^, 347.1344 [M − 162 + H]^+^, 329.1228 [M − 18 − 162 + H]^+^, 215.0913 [M − 132 − 162 + H]^+^, 197.0802 [M − 132 − 162 − 18 + H]^+^, 179.0701 [M − 132 − 162 − 18 − 18 + H]^+^, 161.0598 [M − 132 − 162 − 18 − 18 − 18 + H]^+^, 151.0771 [M − 132 − 162 − 18 − 18 − 28 + H]^+^, 133.0659 [M − 132 − 162 − 18 − 18 − 28 − 18 + H]^+^, 109.0671 [M − 132 − 162 − 18 − 18 − 18 − 52 + H]^+^	
5	5.68	−	521.1854 [M − H]^−^ 567.1918 [M – H + 46]^−^ 1089.3577 [2M – H + 46]^−^	389.1423 [M − 132 − H]^−^, 227.0908 [M − 132 − 162 − H]^−^, 209.0805 [M − 132 − 162 − 18 − H]^−^, 127.0748 [M − 132 − 162 − 100 − H]^−^, 101.0232	pentosyl loganin
		+	523.2039 [M + H]^+^	391.1595 [M − 132 + H]^+^, 229.1062 [M − 132 − 162 + H]^+^, 211.0961 [M − 132 − 162 − 18 + H]^+^, 197.0802 [M − 132 − 162 − 32 + H]^+^, 179.0701 [M − 132 − 162 − 18 − 32 + H]^+^, 161.0598 [M − 132 − 162 − 18 − 32 − 18 + H]^+^, 151.0394 [M − 132 − 162 − 18 − 32 − 28 + H]^+^, 133.0659 [M − 132 − 162 − 18 − 32 − 18 − 28 + H]^+^, 109.0650 [M − 132 − 162 − 18 − 32 − 18 − 52 + H]^+^	
6	5.73	−	357.1183 [M − H]^−^ 403.1223 [M – H + 46]^−^ 761.1247 [2M – H + 46]^−^	195.0650 [M − 162 − H]^−^, 149.0438 [M − 162 − 18 − 28 − H]^−^, 125.0241 [M − 132 − 162 − 70 − H]^−^, 101.0232	sweroside
		+	359.1347 [M + H]^+^ 717.2608 [2M + H]^+^	197.0802 [M − 162 + H]^+^, 179.0701 [M − 162 − 18 + H]^+^, 151.0771 [M − 162 − 18 − 28 + H]^+^, 127.0409 [M − 162 − 70+H]^+^, 109.0671 [M − 162 − 18 − 28 − 42 + H]^+^	
7	5.73	−	389.1342 [M − H]^−^ 435.1502 [M – H + 46]^−^ 793.2800 [2M – H + 46]^−^	227.0939 [M − 162 − H]^−^, 209.0805 [M − 162 − 18 − H]^−^, 197.0797 [M − 162 − 32 − H]^−^, 149 [M − 162 − 18 − 32 − 28 − H]^−^, 131.0356 [M − 162 − 18 − 32 − 28 − 18 − H]^−^, 101.0232	loganin
		+	391.1554 [M + H]^+^ 749.2858 [2M + H]^+^	373.1504 [M − 18 + H]^+^, 229.1062 [M − 162 + H]^+^, 211.0961 [M − 162 − 18 + H]^+^, 179.0701 [M − 162 − 18 − 32 + H]^+^, 151.0771 [M − 162 − 18 − 32 − 28 + H]^+^, 133.0659 [M − 162 − 18 − 32 − 28 − 18 + H]^+^	
8	5.93	−	489.1630 [M − H]^−^ 535.1502 [M − H + 46]^−^ 1025.3314 [2M – H + 46]^−^	195.0650 [M − 132 − 162 − H]^−^, 125.0241 [M − 132 − 162 − 70 − H]^−^, 101.0232	pentosyl sweroside
		+	491.1775 [M + H]^+^	359.1347 [M − 132 + H]^+^, 197.0802 [M − 132 − 162 + H]^+^, 179.0701 [M − 132 − 162 − 18 + H]^+^, 151.0746 [M − 132 − 162 − 18 − 28 + H]^+^, 127.0409 [M − 132 − 162 − 70+H]^+^, 109.0671 [M − 132 − 162 − 18 − 28 − 42 + H]^+^	
9	6.15	−	521.1854 [M − H]^−^ 567.1918 [M – H + 46]^−^ 1043.3804 [2M − H]^−^ 1089.3577 [2M – H + 46]^−^	389.1342 [M − 132 − H]^−^, 371.0636 [M − 132 − 18 − H]^−^, 227.0939 [M − 132 − 162 − H]^−^, 209.0805 [M − 132 − 162 − 18 − H]^−^, 127.0748 [M − 132 − 162 − 100 − H]^−^, 101.0232	loganin 7-*O*-pentoside
		+	523.2039 [M + H]^+^ 1045.4009 [2M + H]^+^	505.1936 [M − 18 + H]^+^, 391.1595 [M − 132 + H]^+^, 373.1504 [M − 18 − 132 + H]^+^, 361.1494 [M − 162 + H]^+^, 343.1367 [M − 18 − 162 + H]^+^, 229.1062 [M − 132 − 162 + H]^+^, 211.0961 [M − 132 − 162 − 18 + H]^+^, 197.0802 [M − 132 − 162 − 32 + H]^+^, 179.0701 [M − 132 − 162 − 32 − 18 + H]^+^, 151.0394 [M − 132 − 162 − 32 − 18 − 28 + H]^+^, 161.0598 [M − 132 − 162 − 32 − 18 − 18 + H]^+^, 133.0659 [M − 132 − 162 − 32 − 18 − 18 − 28 + H]^+^, 109.0650 [M − 132 − 162 − 32 − 18 − 18 − 52 + H]^+^	
10	6.54	−	435.1502 [M – H + 46]^−^ 793.2800 [2M – H + 46]^−^	227.0939 [M − 162 − H]^−^, 209.0805 [M − 162 − 18 − H]^−^, 197.0797 [M − 162 − 32 − H]^−^, 149 [M − 162 − 18 − 32 − 28 − H]^−^, 131.0356 [M − 162 − 18 − 32 − 28 − 18 − H]^−^, 101.0232	7-*epi*-loganin
		+	391.1554 [M + H]^+^ 749.2858 [2M + H]^+^	373.1504 [M − 18 + H]^+^, 229.1062 [M − 162 + H]^+^, 211.0961 [M − 162 − 18 + H]^+^, 179.0701 [M − 162 − 18 − 32 + H]^+^, 151.0771 [M − 162 − 18 − 32 − 28 + H]^+^, 133.0659 [M − 162 − 18 − 32 − 28 − 18 + H]^+^	
11	6.58	−	403.1263 [M − H]^−^ 807.2567 [2M − H]^−^	371.0952[M − 32 − H]^−^, 333.0848 [M − 42 − 28 − H]^−^, 223.0622 [M − 162 − 18 − H]^−^, 191.0354 [M − 162 − 18 − 32 − H]^−^, 165.0565 [M − 162 − 18 − 32 − 28 − H]^−^, 121.0288 [M − 162 − 18 − 32 − 28 − H]^−^, 101.0232	secoxyloganin
		+	405.1383 [M + H]^+^	373.1504 [M − 32 + H]^+^, 345.0262 [M − 32 − 28 + H]^+^, 243.0849 [M − 162 + H]^+^, 225.0774 [M − 162 − 18 + H]^+^, 211.0961 [M − 162 − 32 + H]^+^, 193.0862 [M − 162 − 18 − 32 + H]^+^, 167.0700 [M − 162 − 18 − 32 − 26 + H]^+^, 165.0562 [M − 162 − 18 − 32 − 28 + H]^+^, 127.0380 [M − 32 − 28 − 28 − 28 + H]^+^, 123.0450 [M − 162 − 18 − 32 − 26 − 44 + H]^+^	
12	6.78	−	521.1854 [M − H]^−^ 567.1918 [M – H + 46]^−^ 1043.3804 [2M − H]^−^ 1089.3577 [2M – H + 46]^−^	389.1342 [M − 132 − H]^−^, 371.0636 [M − 132 − 18 − H]^−^, 227.0939 [M − 132 − 162 − H]^−^, 209.0805 [M − 132 − 162 − 18 − H]^−^, 101.0232	7-*epi*-loganin 7-*O*-pentoside
		+	523.2039 [M + H]^+^ 1045.4009 [2M + H]^+^	505.1936 [M − 18 + H]^+^, 391.1595 [M − 132 + H]^+^, 373.1504 [M − 18 − 132 + H]^+^, 361.1494 [M − 162 + H]^+^, 343.1367 [M − 18 − 162 + H]^+^, 229.1062 [M − 132 − 162 + H]^+^, 211.0961 [M − 132 − 162 − 18 + H]^+^, 197.0802 [M − 132 − 162 − 32 + H]^+^, 179.0701 [M − 132 − 162 − 18 − 32 + H]^+^, 151.0394 [M − 132 − 162 − 18 − 32 − 28 + H]^+^, 161.0598 [M − 132 − 162 − 18 − 32 − 18 + H]^+^, 133.0659 [M − 132 − 162 − 18 − 32 − 18 − 28 + H]^+^, 109.0650 [M − 132 − 162 − 18 − 32 − 18 − 52 + H]^+^	
13	6.89	−	387.1302 [M − H]^−^ 433.1331 [M – H + 46]^−^	225.0781 [M − 162 − H]^−^, 179.0540 [M − 162 − 18 − 28 − H]^−^, 155.0347 [M − 162 − 18 − 28 − 24 − H]^−^, 123.0456 [M − 162 − 18 − 28 − 24 − 32 − H]^−^, 101.0232	secologanin
		+	389.1433 [M + H]^+^	227.0920 [M − 162 + H]^+^, 209.0799 [M − 162 − 18 + H]^+^, 195.0666 [M − 162 − 32 + H]^+^, 177.0557 [M − 162 − 18 − 32 + H]^+^, 165.0562 [M − 162 − 18 − 44 + H]^+^, 151.0394 [M − 162 − 18 − 32 − 26 + H]^+^, 149.0599 [M − 162 − 18 − 32 − 28 + H]^+^, 139.0399 [M − 162 − 32 − 28 − 28 + H]^+^, 109.0308 [M − 162 − 18 − 32 − 26 − 42 + H]^+^, 107.0513 [M − 162 − 18 − 32 − 28 − 42 + H]^+^	

Main signals are underlined.

**Table 2:** Characteristics of monoisotopic ion in negative mode and chemical structures of loganic acid 7-*O*-pentoside, *epi*-loganic acid 7-*O*-pentoside, loganic acid, *epi*-loganic acid, and their possible fragments.

Monoisotopic Ion [M − H]^−^ *m*/*z*		Plausible Structure		
Found Calculated Error	Formula			
507.1746 507.17137 *−0.00323*	C_21_H_31_O_14_	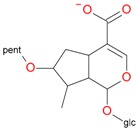		
375.1276 375.12912 *0.00152*	C_16_H_23_O_10_	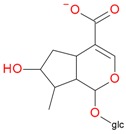		
357.1144 357.11855 *0.00415*	C_16_H_21_O_9_	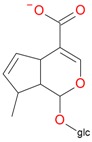		
345.1122 345.11855 *0.00635*	C_15_H_21_O_9_	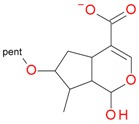		
327.1092 327.10799 *−0.00121*	C_15_H_19_O_8_	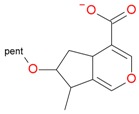		
213.0769 213.07629 *−0.00069*	Aglycone C_10_H_13_O_5_	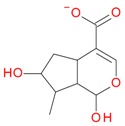	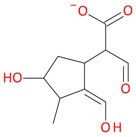	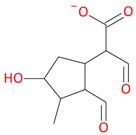
195.0650 195.06573 *0.00073*	C_10_H_11_O_4_	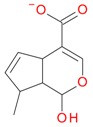	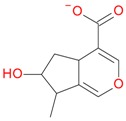	
177.0549 177.05517 *0.00027*	C_10_H_9_O_3_	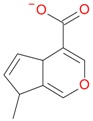		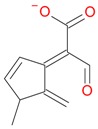
169.0855 169.08646 *0.00096*	C_9_H_13_O_3_	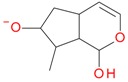	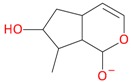	
151.0771 151.0759 −*0.0012*	C_9_H_11_O_2_	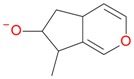	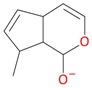	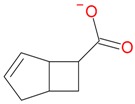
133.0654 133.06534 −*0.00006*	C_9_H_9_O	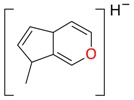	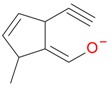	
125.0608 125.06025 −*0.00055*	C_7_H_9_O_2_	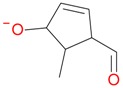	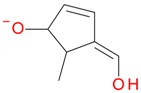	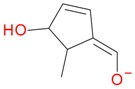
		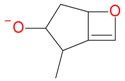	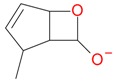	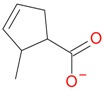
107.0494 107.04969 *0.00029*	C_7_H_7_O		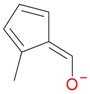	
179.0522 161.0444 143.0344 149.0438 131.0356 119.0341 113.0238 101.0232		Glucose/pentose fragments: 179 (−18) → 161 (−18) → 143 (−18) → 125 149 (−18) → 131 (−18) → 113 119 (−18) → 101		

pent, pentose; glc, glucose.

**Table 3:** Characteristics of monoisotopic ion in positive mode and chemical structures of loganic acid 7-*O*-pentosideside, *epi*-loganic acid 7-*O*-pentoside, loganic acid, *epi*-loganic acid, and their possible fragments.

Monoisotopic Ion [M + H]^+^ *m*/*z*		Plausible Structure		
Found Calculated Error	Formula			
509.1876 509.18702 *−0.00058*	C_21_H_33_O_14_	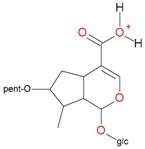	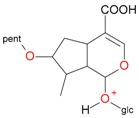	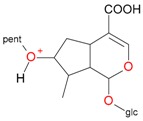
491.1775 491.17646 *−0.00104*	C_21_H_31_O_13_	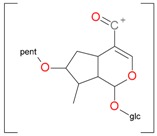	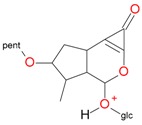	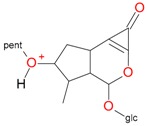
377.1493 377.14477 *−0.00453*	C_16_H_25_O_10_	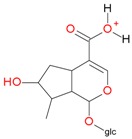	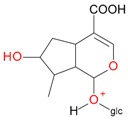	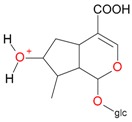
359.1348 359.1342 *−0.0006*	C_16_H_23_O_9_	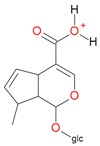	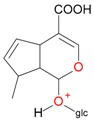	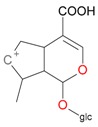
		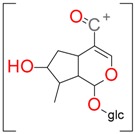	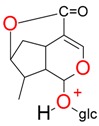	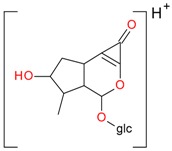
347.1344 347.1342 *−0.0002*	C_15_H_23_O_9_	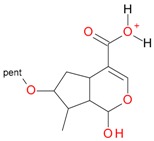	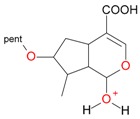	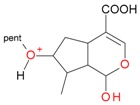
329.1228 329.12364 *0.00084*	C_15_H_21_O_8_	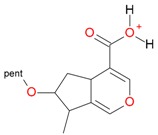	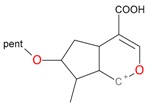	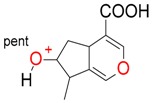
		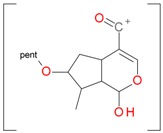	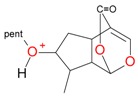	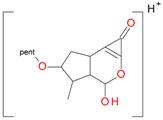
215.0919 215.09194 *0.00004*	Aglycone C_10_H_15_O_5_	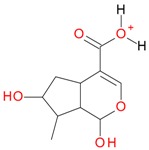	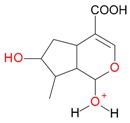	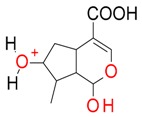
197.0816 197.08138 *−0.00022*	C_10_H_13_O_4_	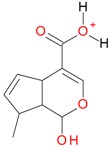	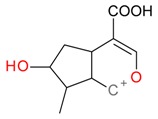	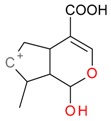
		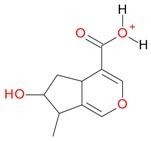	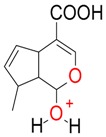	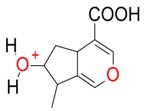
		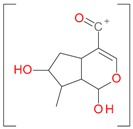	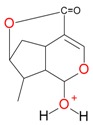	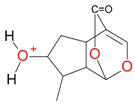
			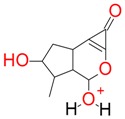	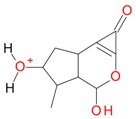
179.0721 179.07082 *−0.00128*	C_10_H_11_O_3_	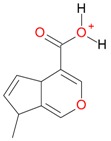	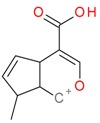	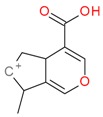
		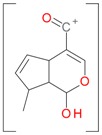	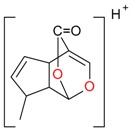	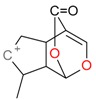
		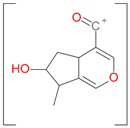	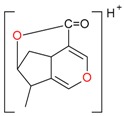	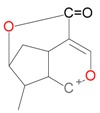
			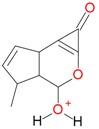	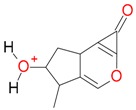
161.0598 161.06025 *0.00045*	C_10_H_9_O_2_	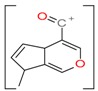	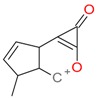	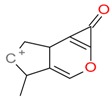
			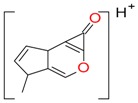	
151.0771 151.0759 *−0.0012*	C_9_H_11_O_2_		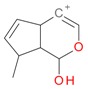	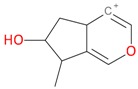
			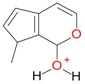	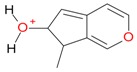
133.0659 133.06534 *−0.00056*	C_9_H_9_O	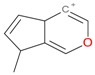	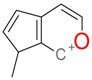	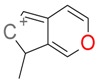
			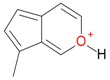	
109.0671 109.06534 *−0.00176*	C_7_H_9_O	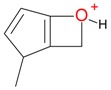		

pent, pentose; glc, glucose.

**Table 4:** Characteristics of monoisotopic ion in negative mode and chemical structures of pentosyl loganin, loganin 7-*O*-pentoside, 7-*epi*-loganin 7-*O*-pentoside, loganin, and 7-*epi*-loganin, and their possible fragments.

Monoisotopic Ion [M − H]^−^ *m/z*		Plausible Structure		
Found Calculated Error	Formula			
**567.1918** [M + FA − H]^−^ 567.1925 *0.0007* **521.1854** 521.18702 *0.00162*	C_22_H_33_O_14_ CH_2_O_2_	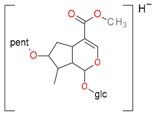	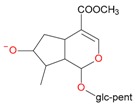	
**435.1502** [M + FA − H]^−^ 435.15024 *0.00004* **389.1423** 389.14477 *0.00247*	C_17_H_25_O_10_ CH_2_O_2_	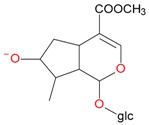		
**359.1379** 359.1342 *−0.0037*	C_16_H_23_O_9_	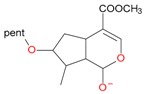		
**227.0908** 227.09194 *0.00114*	Aglycone C_11_H_15_O_5_	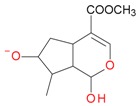	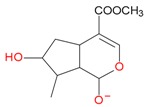	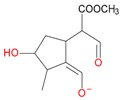
	C_10_H_13_O_3_ CH_2_O_2_	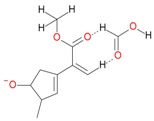	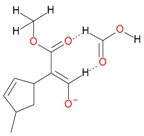	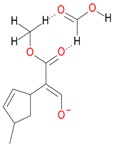
**209.0805** 209.08138 *0.00088*	C_11_H_13_O_4_	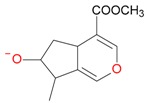	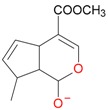	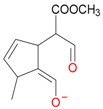
**127.0748** 127.0759 *0.0011*	C_7_H_11_O_2_	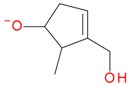	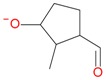	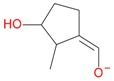
**101.0252** 101.02387 *−0.00133*	C_4_H_5_O_3_	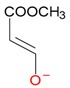		
**179.0540** **161.0449** **143.0368** **149.0448** **131.0333** **119.0354** **113.0238** **101.0268**		Glucose/pentose fragments: 179 (−18) → 161 (−18) → 143 (−18) → 125 149 (−18) → 131 (−18) → 113 119 (−18) → 101		

pent, pentose; glc, glucose.

**Table 5:** Characteristics of monoisotopic ion in positive mode and chemical structures of pentosyl loganin, loganin 7-*O*-pentoside, 7-*epi*-loganin 7-*O*-pentoside, loganin, and 7-*epi*-loganin, and their possible fragments.

Monoisotopic Ion [M + H]^+^ *m/z*		Plausible Structure		
Found Calculated Error	Formula			
**523.2039** 523.20267 *−0.00123*	C_22_H_35_O_14_	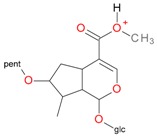	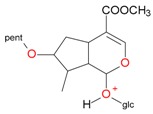	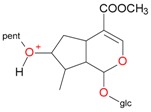
		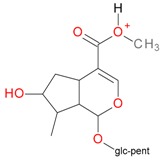	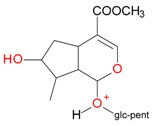	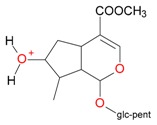
**505.1936** 505.19211 *−0.00149*	C_22_H_33_O_13_	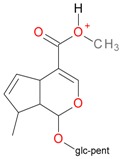	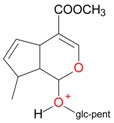	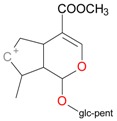
**391.1595** 391.16041 *0.00091*	C_17_H_27_O_10_	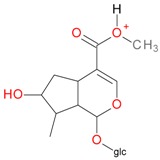	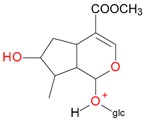	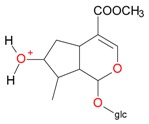
**373.1504** 373.14985 *−0.00055*	C_17_H_25_O_9_	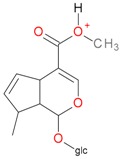	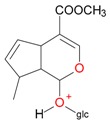	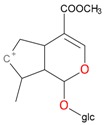
**361.1494** 361.14985 *−0.00045*	C_16_H_25_O_9_	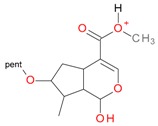	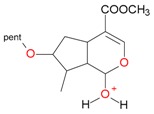	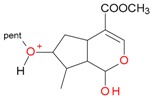
**343.1367** 343.13929 *0.00299*	C_16_H_23_O_8_	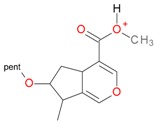	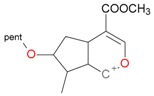	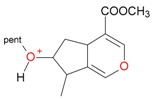
**229.1062** 229.10759 *0.00259*	Aglycone C_11_H_17_O_5_	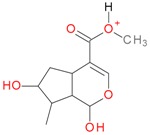	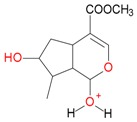	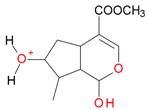
**211.0961** 197.09703 *−0.00093*	C_11_H_15_O_4_	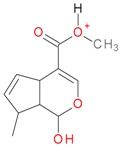	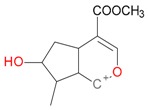	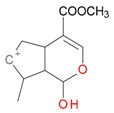
		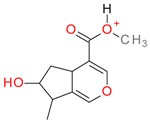	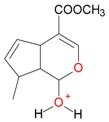	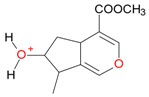
**197.0802** 197.08138 *0.000118*	C_10_H_13_O_4_	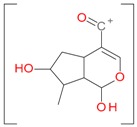	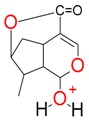	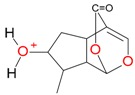
			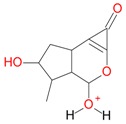	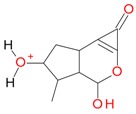
**179.0701** 179.07082 *0.00072*	C_10_H_11_O_3_	See Table 3		
**161.0598** 161.06025 *0.00045*	C_10_H_9_O_2_			
**151.0771** 151.0759 *−0.0012*	C_9_H_11_O_2_			
**133.0659** 133.06534 *−0.00056*	C_9_H_9_O			
**109.0650** 109.06534 *0.00034*	C_7_H_9_O			

pent, pentose; glc, glucose.
